# Supplementary material for: Predictors of health self-management behaviour in Kazakh patients with metabolic syndrome: A cross-sectional study in China
Source: PLoS One. 2022 Dec 20;17(12):e0278190. doi: 10.1371/journal.pone.0278190 (PMC9767334; doi:10.1371/journal.pone.0278190)
Supplement: S1 Table — (DOCX) [file pone.0278190.s001.docx]

Table S1. Correlation analysis of health self-management behaviour with metabolic indicators (*n*=454)

| Variables | Mean ± SD  (female) | Mean ± SD  (male) | Mean ± SD  (total) | Diet | Exercise | Other lifestyles | Medication | Disease self-monitoring | Emotion | Communication with physicians | Overall SMB |
| --- | --- | --- | --- | --- | --- | --- | --- | --- | --- | --- | --- |
| Weight | 70.95±12.33 | 84.61±12.25 | 77.48±14.05 | 0.006 | 0.003 | -0.017 | -0.042 | 0.066 | -0.028 | 0.111^*^ | 0.018 |
| WC | 90.27±9.33 | 95.72±9.46 | 92.87±9.77 | -0.058 | 0.046 | 0.009 | -0.059 | 0.014 | 0.007 | 0.059 | -0.010 |
| BMI | 28.30±4.46 | 28.68±3.49 | 28.48±4.03 | 0.026 | 0.015 | -0.057 | -0.072 | 0.049 | -0.044 | 0.004 | -0.023 |
| SBP | 135.58±11.31 | 137.93±10.65 | 136.71±11.05 | -0.053 | -0.023 | -0.061 | -0.104^*^ | -0.018 | -0.023 | 0.087 | -0.058 |
| DBP | 88.90±6.41 | 90.10±6.67 | 89.47±6.56 | -0.056 | -0.026 | -0.060 | -0.130^**^ | 0.043 | 0.018 | 0.041 | -0.054 |
| FPG | 5.13±1.10 | 4.98±1.04 | 5.06±1.07 | -0.004 | 0.002 | -0.016 | 0.063 | 0.048 | -0.056 | -0.054 | -0.011 |
| TC | 5.55±1.21 | 5.59±1.10 | 5.57±1.16 | -0.073 | -0.020 | -0.008 | -0.023 | -0.047 | -0.025 | -0.050 | -0.071 |
| TG | 1.76±1.22 | 2.09±1.10 | 1.92±1.17 | 0.031 | -0.026 | 0.057 | -0.051 | -0.060 | 0.042 | 0.006 | 0.015 |
| HDL-C | 1.31±0.35 | 1.23±0.37 | 1.27±0.36 | -0.054 | -0.068 | -0.065 | -0.010 | 0.098^*^ | -0.031 | 0.028 | -0.040 |
| LDL-C | 2.88±1.03 | 3.17±0.99 | 3.02±1.02 | -0.047 | -0.032 | -0.021 | 0.057 | -0.054 | -0.038 | 0.011 | -0.036 |

Note: SMB, self-management behaviour; SD, standard deviation; WC, waist circumference; BMI, Body mass index; SBP, systolic blood pressure; DBP, diastolic blood pressure; FPG, fasting plasma glucose; TC, total cholesterol; TG, triglyceride; HDL-C, high density lipoprotein cholesterol; LDL-C, low density lipoprotein cholesterol;**, *P*＜0.01; *, *P*＜0.05.
