# Supplementary material for: Predictors of health self-management behaviour in Kazakh patients with metabolic syndrome: A cross-sectional study in China
Source: PLoS One. 2022 Dec 20;17(12):e0278190. doi: 10.1371/journal.pone.0278190 (PMC9767334; doi:10.1371/journal.pone.0278190)
Supplement: S2 Table — (DOCX) [file pone.0278190.s002.docx]

Table S2. Correlation analysis of health self-management behaviour with knowledge of MS, self-efficacy and social support (*n*=454)

| Variables | Median  （P25，P75） | Diet management | Exercise management | Other lifestyles management | Medication management | Disease self-monitoring | Emotion management | Communication with physicians | Overall SMB |
| --- | --- | --- | --- | --- | --- | --- | --- | --- | --- |
| **Knowledge of MS** | 0.00 (0.00, 10.00) | 0.010 | -0.172^**^ | 0.154^**^ | 0.102^*^ | -0.102^*^ | -0.044 | -0.026 | 0.024 |
| Definition of MS | 0.00 (0.00, 0.00) | -0.010 | -0.125** | 0.143** | 0.074 | -0.108* | -0.077 | -0.025 | -0.007 |
| Relationship between MS and CVD | 0.00 (0.00, 0.00) | -0.016 | -0.127** | 0.133** | 0.063 | -0.079 | -0.147** | -0.066 | -0.036 |
| Prevention of MS | 0.00 (0.00, 10.00) | -0.002 | -0.245** | 0.142** | 0.054 | -0.089 | -0.063 | -0.046 | -0.005 |
| **Self-Efficacy** | 4.08 (3.00, 5.33) | 0.014 | -0.042 | -0.016 | 0.096^*^ | 0.040 | -0.048 | 0.010 | 0.027 |
| Symptom management | 4.00 (2.50, 5.00) | -0.005 | -0.056 | -0.021 | 0.064 | 0.039 | -0.040 | -0.007 | -0.005 |
| General disease management | 4.50 (3.00, 3.00) | 0.026 | 0.005 | -0.021 | 0.098* | 0.041 | -0.037 | 0.016 | 0.049 |
| **Social Support** | 40.00 (37.00, 45.00) | -0.020 | -0.033 | 0.041 | -0.038 | -0.061 | -0.066 | 0.008 | -0.036 |
| Subjective support | 24.00 (2.00, 27.00) | 0.000 | -0.057 | -0.001 | -0.029 | -.0032 | 0.103* | -0.055 | -0.061 |
| Objective support | 10.00 (9.00, 12.00) | -0.012 | 0.007 | -0.017 | 0.035 | -0.015 | -0.019 | 0.024 | -0.012 |
| Utilization of support | 6.00 (5.00, 9.00) | 0.008 | 0.022 | 0.097* | -0.040 | -0.049 | 0.002 | 0.087 | 0.043 |

Note: SMB, self-management behaviour; **, *P*＜0.01; *, *P*＜0.05.
