# Supplementary material for: Predictors of health self-management behaviour in Kazakh patients with metabolic syndrome: A cross-sectional study in China
Source: PLoS One. 2022 Dec 20;17(12):e0278190. doi: 10.1371/journal.pone.0278190 (PMC9767334; doi:10.1371/journal.pone.0278190)
Supplement: S3 Table — (DOCX) [file pone.0278190.s003.docx]

Table S3. Variables and the assignments of logistic regression analysis

| Variables | Assignments |
| --- | --- |
| Y: self-management behaviour, diet management, exercise management, other lifestyle management, medication management, disease self-monitoring, emotion management, and communication with physicians | good=0, poor=1  Self-management behaviour was divided into two groups using the score index: <60% indicates “poor”, and ≥60% indicates “good”. The index scoring = (actual total score/possible highest score)×100%. |
| X1: Sex | male=0, female=1 |
| X2: Age | Original value |
| X3: Education | illiteracy=1, elementary school=2,  middle school=3, high school and above=4 |
| X4: Marital Status | partnered=0, un-partnered=1 |
| X5: Occupation | non-agriculture and animal husbandry =0, Agriculture and animal husbandry=1 |
| X6: Living status | live with others=0, live alone=1 |
| X7: Place of residence | cities and towns=0, pastoral area=1 |
| X8: Income (yuan/month/person) | ≤1000 yuan=1, 1001~3000 yuan=2, 3001~5000 yuan=3, ≥5000 yuan=4 |
| X9: Method of paying medical expenses | urban medical insurance=0, private expense =1 |
| X10: Chronic disease comorbidities | no=0, yes=1 |
| X11: Family heredity history | no=0, yes=1 |
| X12: Number of MS components | Original value |
| X13: Weight | Original value |
| X14: Body mass index (BMI) | Original value |
| X15: Waist circumference (WC) | Original value |
| X16: Systolic blood pressure (SBP) | Original value |
| X17: Diastolic blood pressure (DBP) | Original value |
| X18: Total cholesterol (TC) | Original value |
| X19: Triglyceride（TG） | Original value |

Table S3 (continued). Variables and the assignments of logistic regression analysis

| Variables | Assignments |
| --- | --- |
| X20: High density lipoprotein cholesterol (HDL-C) | Original value |
| X21: Low density lipoprotein cholesterol (LDL-C) | Original value |
| X22: Fasting plasma glucose (FPG) | Original value |
| X23: Knowledge of MS | Original value |
| X23a: Definition of MS | Original value |
| X23b: Relationship between MS and CVD | Original value |
| X23c: Prevention of MS | Original value |
| X24: Self-efficacy | Original value |
| X24a: Symptom management | Original value |
| X24b: General disease management | Original value |
| X25: Social support | Original value |
| X25a: Subjective support | Original value |
| X25b: Objective support | Original value |
| X25c: Utilization of support | Original value |
