# Supplementary material for: Predictors of health self-management behaviour in Kazakh patients with metabolic syndrome: A cross-sectional study in China
Source: PLoS One. 2022 Dec 20;17(12):e0278190. doi: 10.1371/journal.pone.0278190 (PMC9767334; doi:10.1371/journal.pone.0278190)
Supplement: Attachment — Submitted filename: Review_PONE-D-20-30645_Josiane Bonnefoy.pdf [file pone.0278190.s004.pdf]

Review:
'Predictors of health self-management behaviour in Kazakh patients with metabolic syndrome: a cross-sectional study in China'

In my opinion, this is a well-developed paper. The authors present clearly the problem addressed and go thoroughly through it. I also consider that it represents a contribution in terms of incorporating culturally specific issues that should be taken into account when designing health interventions.

I have the following comments, which I consider important to improve in this paper.

1. The 'Other lifestyle management" dimension is a broad category. Since it is significant in different areas, e.g., education, I would suggest giving a couple of examples to illustrate what is included under 'other lifestyle management'.

2. 'Communication with physicians': I would suggest to specify whether Kazakh people speak their own language, or whether they are linguistically integrated. Since more than 50% of the participants registered a low educational level (primary school education or illiterate), this could be exacerbated by language issues. If so, this issue should also be taken into account in conducting patient health self-management interventions.

3. [Lines 235-240] Would it be possible to include some information which may explain these results ('moderate level', 'upper-middle level', 'good level') in other ethnic groups? In order to understand what is it about those studies that show different results.

4. The authors state at the beginning that Kazakh people are Muslims, but this dimension is not addressed in the paper.

   Besides the arguments given on how traditional Kazakh culture affects health self-management behaviour, it would be useful to know whether there are factors which may be directly related with their religion, e.g., diet. It might be easier to change other areas which may affect HSM, but religious belief that guide daily life are more difficult to address, and therefore it is relevant to identify them; for example, the Lundberg study (cited in lines 242-245): 'Muslim women with type 2 diabetes reported that it was very difficult to change their lifestyles and perform health self-management according to the advice of medical staff''.

5. Though the authors declare that their methodological approach will be based on univariate analysis, in order to fully understand the impact of the three dimensions, it would be necessary to present data on the interactions among gender (1), education (2), and family monthly income per capita (3). Or at least between gender and education.

   Secondly, how these three variables interact with 'Knowledge of MS' (4.2.3), 'Self-efficacy (4.2.4)', and 'Social support' (4.2.5)

   Otherwise, we don`t know whether women have poor communication with doctors just because they are women or because they also have lower educational levels than men.

6. The analysis undergone in the Social support section (4.2.5) is quite well accomplished since it examines the cultural causes and how they relate, as well as explaining its relationship with health self-management behaviour .

7. [Lines 420-421] '...researchers should consider the effects of cultural differences and disease characteristics on health self-management behaviour based on the health belief model'.

   It is quite valuable that the authors conclude highlighting the effects of cultural differences, but precisely to do so, authors should be more specific on the points suggested above.

8. Though I recognise that it is beyond the authors' methodological scope, I would suggest presenting at least one DAG, which will allow greater understanding when designing interventions, since it would illustrate interactions among the different studied variables.

9. [Line 159] 'mean BMI of the participants was 8.48'. Is it 28.4 instead of 8.4?

10. Reference 23:

    23. Jaqueline, Vicente E, María Isabel, et al. Cuestionario de asertividad centrado en el estilo de vida CACEV) en pacientes con sindrome metabolico: desarrolloy validacion. Behavioral Psychology /Psicología Conductual. 2017;25(2):349-69.

    (a) There is a confusion with the Spanish names and surnames:

    The correct way is: Garcia-Silva J, Caballo VE, Peralta-Ramírez MI, Lucena-Santos P, Navarrete-Navarrete N.

    --> Garcia-Silva J, Caballo VE, Peralta-Ramírez MI, et al.
    (b) 'desarrolloy validacion' should say: desarrollo y validacion
