# Supplementary material for: Predictors of health self-management behaviour in Kazakh patients with metabolic syndrome: A cross-sectional study in China
Source: PLoS One. 2022 Dec 20;17(12):e0278190. doi: 10.1371/journal.pone.0278190 (PMC9767334; doi:10.1371/journal.pone.0278190)
Supplement: Attachment — Submitted filename: Response to Reviewers.docx [file pone.0278190.s005.docx]

**Response to Reviewers**

Dear Editors and Reviewers:

We would like to thank you for your kind letter and for the reviewers’ constructive comments concerning our manuscript entitled “Predictors of health self-management behaviour in Kazakh patients with metabolic syndrome: a cross-sectional study in China” (Manuscript No.:PONE-D-20-30645). All the comments were valuable and highly helpful in revising and improving our paper as well as ensuring the significance of our research was clear. All the authors seriously discussed all the comments. According to the reviewers’ comments, we have made corrections that we hope will be met with approval. The revised sections are marked in red in the paper. The main corrections in the paper and our responses to the reviewer’s comments are as follows:

Responds to the reviewer’s comments:

Reviewer #1:

1. Comment: “suggest giving a couple of examples to illustrate what is included under ‘other lifestyle management’”.

Response:

We apologize for neglecting the description of the category of "other lifestyle management" in the manuscript. We have now provided a supplementary explanation in lines 110-111.

2. Comment: “suggest to specify whether Kazakh people speak their own language, or whether they	are linguistically integrated”.

Response:

Considering the reviewer’s suggestion, we have provided specific explanations in lines 300-304.

3. Comment: “[Lines 235-240]: explain these results (‘moderate level’, ‘upper-middle level’, ‘good level’) in other ethnic groups”.

Response:

Considering the reviewer’s suggestion, we have provided the explanations in lines 241-245.

4. Comment: “it would be useful to know whether there are factors which may be directly related with their religion, e.g., diet”.

Response:

As the reviewer suggested, the health self-management behaviour of Kazakh MS patients is directly related to their religious beliefs, which are explained in lines 409-412. Additionally, we explain the correlation with diet and other aspects in lines 253-258.

5. Comment: “suggest to present data on the interactions among gender, education, and family monthly income per capita”.

Response:

Thank you very much for your suggestion. It is in fact necessary to further explore the interaction of various influencing factors on health self-management behaviour. However, due to the limited length of the article, only univariate analysis and multivariate logistic regression analysis were conducted. We intend to further analyse this topic elsewhere (i.e., in research on the influencing mechanism of health self-management behaviour in Kazakh patients with metabolic syndrome).

In addition, the reasons for “women have poor communication with doctors” are explained in lines 275-282.

6. Comment: “The analysis undergone in the Social support section (4.2.5) is quite well accomplished since it examines the cultural causes and how they	relate, as well as explaining its relationship	with health self-management behaviour”.

Response:

Thank you very much for this appreciative comment.

7. Comment: “consider making more specific suggestions on cultural differences”.

Response:

This problem is described in lines 430-434.

8. Comment: “suggest presenting at least one DAG”.

Response:

Thank you very much for your advice. In response to your suggestion, we will consider presenting DAG in another study (i.e., in research on the influencing mechanism of health self-management behaviour in Kazakh patients with metabolic syndrome) to better clarify the interactions among the different studied variables.

9. Comment: “[Line 159]	‘mean BMI of the participants	was	8.48’. Is	it 28.4 instead of 8.4?”.

Response:

We sincerely regret our careless error. Thank you very much for noting this matter. We have corrected the mistake according to your suggestions.

10. Comment: “Reference 23, there is a confusion with the Spanish names and surnames”.

Response:

Thank you very much for your comment. We have amended the text accordingly.

Reviewer #2:

Comment: “The article presents a very important and relevant topic for public health. I really liked the methodology and discussion. My only suggestion is to improve the clinical and practical implications of the study”.

Response:

Thank you very much for your positive comments on our manuscript. We have tried our best to improve the manuscript according to your suggestion.

We earnestly appreciate the editors/reviewers’ sincere feedback and hope that our corrections will meet with their approval. PLOS One is an influential, highly informative journal, and we are grateful to have our article considered for publication there. Should additional corrections be required, please let us know.
