# Supplementary material for: Predictors of health self-management behaviour in Kazakh patients with metabolic syndrome: A cross-sectional study in China
Source: PLoS One. 2022 Dec 20;17(12):e0278190. doi: 10.1371/journal.pone.0278190 (PMC9767334; doi:10.1371/journal.pone.0278190)
Supplement: Attachment — Submitted filename: Response to Reviewers.docx [file pone.0278190.s007.docx]

**Response to Reviewers**

Dear Editors and Reviewers:

We would like to thank you for your kind letter and for the reviewers’ constructive comments concerning our manuscript entitled “Predictors of health self-management behaviour in Kazakh patients with metabolic syndrome: A cross-sectional study in China” (Manuscript Number: PONE-D-20-30645R1). All the comments were valuable and highly helpful in revising and improving our paper as well that ensuring that the significance of our research was clear. All the authors seriously discussed all the comments. According to the reviewers’ comments, we have made changes that we hope will meet with your approval. The revised sections are marked in red in the paper. The main corrections in the paper and our responses to the reviewer’s comments are as follows:

**Reviewer #2:**

Comment: “The authors made all the adjustments that were requested. Thus, the air quality greatly improved. Congratulations to the authors for the excellent work that was done”.

Response:

Thank you very much for your positive comments about the further adjustments and improvements made to the manuscript.

**Reviewer #3:**

**Comment 1**: “ABSTRACT: A brief background, plus the aim, should be added.”

Response:

Considering the reviewer’s suggestion, we have added this information to lines 3-7.

**Comment 2:** “INTRODUCTION: Newest Global/ Regional/ China prevalence of metabolic syndrome should be mentioned.”

Response:

Considering the reviewer’s suggestion, we again researched the prevalence of metabolic syndrome and unfortunately did not find the latest reports.

**Comment 3:** “METHODS: Sample size should be explained.”

Response:

Considering the reviewer’s suggestion, we have provided this explanation in lines 86-92.

**Comment 4:** “RESULTS: a) In the comments of table 3, it is advisable to explain the main influencing factors for the total SMB and for each dimension in the table.”

Response:

Thank you very much for your suggestion. Indeed, in addition to reporting the main influencing factors of the overall SMB in a comprehensive manner, a further presentation of the influences of each dimension would make the results clearer. However, due to space constraints, we have shown the influencing factors for each dimension in detail in table 3, so no further textual presentation has been made.

**Comment 4:** “RESULTS: b) Please, revise and redo the regression analysis (table 3) considering the positive and negative influencing factors. For example, for education, the B is -ve and the OR is less than 1, so it is a negative influencing factor not a positive one and so on.”

Response:

Thank you very much for your suggestion. We have checked and analysed this section with care. In logistic regression models, the regression coefficient (B) is logarithmically related to the dominance ratio, so that when the regression coefficient is -ve, it corresponds to an OR<1. The dominance ratio can be used as an indicator to estimate the effect size, which measures the magnitude of the dominant influence of an independent variable, and the significance of the OR value is that when assigning a larger value to the event occurrence group,

(1) OR=1, indicating that OR=1 means that the factor has no effect on the occurrence of the event;

(2) OR>1 means that the factor is a risk factor for the occurrence of the event (negative influence);

(3) OR<1 means that the factor is a protective factor for the occurrence of the event (positive influence).

In this study, we used the total health self-management behaviour score and each dimension score as dependent variables, divided the self-management behaviour into two groups using the score index, and performed logistic regression. We assigned the values of self-management behaviour as the dependent variable as follows: good=0, poor=1, as shown in Table S3, and the final results were obtained (Table 3). As an example of emotion management behaviour, the presence or absence of chronic disease comorbidity (no=0, yes=1); showed that B = 0.630, OR = 1.874 >1, indicating that the risk of poor emotion management behaviour among study participants with chronic comorbidities was 1.874 times higher than that for participants without chronic comorbidities, and that chronic comorbidities were negative influencing factors on emotion management behaviour. For this reason, we did not modify this section.

**Comment 5:** “DISCUSSION: a) It is advisable to explain the study objective at the beginning of the discussion. ”

Response:

Considering the reviewer’s suggestion, we have provided the explanation in lines 237-245.

**Comment 5:** “DISCUSSION: b) It is better to write that any factor has OR less than 1 is less likely to cause health self-management behavior and vice versa. ”

Response:

In conjunction with the explanation of "Comment 4(b)", we did not modify the relevant content.

**Comment 5:** “DISCUSSION: c) The word “Table 3” should not be written in the discussion.”

Response:

Thank you very much for your suggestion. For the completeness of the content and for the convenience of the reader (the content of the supplementary tables are not shown in the text), we have retained the label "Table" in the discussion section.

**Comment 6:** “STRENGTHS AND LIMITATIONS: a) Please, analyze the strengths and limitations of the study.”

Response:

Considering the reviewer’s suggestion, we have added the limitations of the study in lines 454-462.

**Reviewer #4:**

**Comment 1:** “Abstract: Please rephrase the following sentence: ‘The significant positive predictors of health self-management behaviour were gender…’. It is not clear whether male or female was the positive predictor.”

Response:

We apologize for neglecting the sex-specific description in the manuscript. We have now provided a modification in line 227.

**Comment 2:** “Introduction: Please provide references for the following sentences in the Introduction section: 1. ‘The occurrence of MS is related to many factors, such as race, genetics, culture, religious beliefs, and lifestyle.’ 2. “Long-term nomadic life has caused Kazakhs to develop a unique lifestyle and cultural background that is distinct from other ethnic groups, which may be the main reason for their high incidence of MS.”

Response:

Thank you very much for your careful reminder. We have provided the supplementary references in lines 49 and 55.

**Comment 3:** “Methods: Inclusion criteria: should add Kazakhs.”

Response:

Considering the reviewer’s suggestion, we have added Kazakhs to the inclusion criteria in line 79.

**Comment 4:** “Results: There appears to be a discrepancy between the text and Table 1 regarding percentage of women, in the table; 52.2% while in the text; 52.8%.”

Response:

We apologize for the incorrect number, and we have corrected it in line 165.

**Comment 5:** “Results: 159 ‘Overweight and obesity were defined as a BMI≥24.0 kg/m^2^ and a BMI≥28.0 kg/m2, respectively.’, the definition should be in the Methods section.”

Response:

Considering the reviewer’s suggestion, we have moved the definition of overweight and obesity to the methods section in lines 105-106.

**Comment 6:** “Results: Figure 1, Table 3 and Table S1– Please provide the expanded form of the abbreviations (in the figure cation and table note).”

Response:

Considering the reviewer’s suggestion, we have supplemented the expanded form of the abbreviations in Figure 1, Table 3 and Table S1 in lines 187-188, 233-234 and Table S1 in that order.

**Comment 7:** “Results: Lines 208-210: ‘…gender, education, family monthly income per capita, weight, knowledge of MS, and self-efficacy were positive influencing factors of health self-management behaviour…’. It is not clear if female or male was associated positively with self-management. Please rewrite the sentence.”

Response:

Considering the reviewer’s suggestion, we have made this point explicit in line 227 based on the results of the study.

**Comment 8:** “Discussion: -Line 234- ‘The results of the above studies all indicated that the overall level of health…’ needs further editing.”

Response:

Considering the reviewer’s suggestion, we have further edited and improved this sentence in lines 264-266.

**Comment 9:** “Discussion: The following paragraph is not evidence-based (at least not by this paper) and it seems to be not correct. The explanation may relate to nomadic unique lifestyle (and not to the religious beliefs). I suggest to delete or rephrase the following:

‘Here, the explanation may relate to the religious beliefs of the studied ethnic groups. Religious taboos in the Quran, such as those regarding diet, behaviour and certain types of emotional expression, have a profound influence on the Kazakhs who believe in Islam. These taboos not only play an important guiding role with respect to their health concept, lifestyles and social life, but also have a strong restraining effect, which may affect the health self-management behaviour of Kazakh MS patients to a certain extent.’”

Response:

Thank you very much for your suggestion. This section has been revised accordingly in lines 283-284, but the discussion on the influencing factor "religious beliefs" has been retained. The reasons for this are follows: on the one hand, the reviewer suggested adding this section during the first round of peer review; on the other hand, the authors have been living in Xinjiang, China, and have experienced first-hand that religious beliefs do have an impact on the health concepts, lifestyles and social life of this population.

**Comment 10:** “Discussion: There is no need to specify in detail the results in the Discussion section, it should summarize the main points but not duplicate of the Results section. I suggest to minimize the univariate analysis results in the Discussion section. For example, you can minimize the marked paragraph: The univariate analysis showed that gender was not significantly associated with the total health self-management behaviour scores and the scores of each dimension among Kazakh MS patients in China (Table 1). However, gender was included in the multi-factor regression analysis because we considered that gender may have a certain impact on the health self-management behaviour of Kazakh MS patients from a professional perspective. In the logistic regression analysis, being male was a protective factor for communication with physicians in Kazakh MS patients (Table 3).”

Response:

Thank you very much for your suggestion, indeed as you say there is no need to specify in detail the results in the discussion section, which we have summarized in lines 301-302.

**Comment 11:** “Discussion: -Line 294-295: "Education was a risk factor for exercise management behaviour…" Counterintuitive, education was a risk factor? Please note that the OR in table 3 was not significant.”

Response:

Again, we are deeply apologetic for our carelessness and thank you very much for your prompt and careful suggestion. After double-checking the data in Table 3, it was confirmed that the p=0.092 > 0.05 for the influencing factor education was not statistically significant, so we have made a correction in lines 322-323.

**Comment 12:** “In the Discussion section please add the limitations of the study, for example, the use of convenience sampling.”

Response:

Considering the reviewer’s suggestion, we have added the limitations of the study in lines 454-462.

We earnestly appreciate the editors/reviewers’ sincere feedback and hope that our changes will meet with their approval. PLOS One is an influential, highly informative journal, and we are grateful to have our article considered for publication. Should additional corrections be needed, please let us know.
