# Supplementary material for: Predictors of health self-management behaviour in Kazakh patients with metabolic syndrome: A cross-sectional study in China
Source: PLoS One. 2022 Dec 20;17(12):e0278190. doi: 10.1371/journal.pone.0278190 (PMC9767334; doi:10.1371/journal.pone.0278190)
Supplement: Attachment — Submitted filename: Reviewer comments PONE-D-20-30645_R1.docx [file pone.0278190.s006.docx]

Reviewer comments

Manuscript Number: PONE-D-20-30645_R1

Title "Predictors of health self-management behaviour in Kazakh patients with metabolic syndrome: A cross-sectional study in China".

**Generally speaking:**
Thank you for providing me the opportunity to review this manuscript that raises important issues about health self-management behavior in Kazakh patients with metabolic syndrome.

**Comment 1:**

1. *ABSTRACT:*
a) A brief background, plus the aim, should be added.

**Comment 2:**
*2. INTRODUCTION:*

a) Newest Global/ Regional/ China prevalence of metabolic syndrome should be mentioned.

**Comment 3:**
*3. METHODS:*

a) Sample size should be explained.

**Comment 4:**
*4. RESULTS:*

a) In the comments of table 3, it is advisable to explain the main influencing factors for the total SMB and for each dimension in the table.
b) Please, revise and redo the regression analysis (table 3) considering the positive and negative influencing factors. For example, for education, the B is -ve and the OR is less than 1, so it is a negative influencing factor not a positive one and so on.

**Comment 5:**

*5. DISCUSSION:*

a) It is advisable to explain the study objective at the beginning of the discussion.
b) It is better to write that any factor has OR less than 1 is less likely to cause health self-management behavior and vice versa.
c) The word “Table 3” should not be written in the discussion.

**Comment 6:**

*6. STRENGTHS AND LIMITATIONS:*

a) Please, analyze the strengths and limitations of the study.
