# Supplementary material for: Predictors of health self-management behaviour in Kazakh patients with metabolic syndrome: A cross-sectional study in China
Source: PLoS One. 2022 Dec 20;17(12):e0278190. doi: 10.1371/journal.pone.0278190 (PMC9767334; doi:10.1371/journal.pone.0278190)
Supplement: Attachment — Submitted filename: Reviewer comments PONE-D-20-30645_R2.docx [file pone.0278190.s008.docx]

Reviewer comments

Manuscript Number: PONE-D-20-30645_R2

Title "Predictors of health self-management behaviour in Kazakh patients with metabolic syndrome: A cross-sectional study in China".

Thank you for providing me the opportunity to re-review this manuscript that raises important issues about Predictors of health self-management behavior in Kazakh patients with metabolic syndrome: A cross-sectional study in China.

It seems that all corrections were done.
